# Gene Expression in Osteolysis: Review on the Identification of Altered Molecular Pathways in Preclinical and Clinical Studies

**DOI:** 10.3390/ijms18030499

**Published:** 2017-02-25

**Authors:** Francesca Veronesi, Matilde Tschon, Milena Fini

**Affiliations:** Laboratory of Preclinical and Surgical Studies, Rizzoli Orthopedic Institute, via di Barbiano 1/10, 40136 Bologna, Italy; francesca.veronesi@ior.it (F.V.); milena.fini@ior.it (M.F.)

**Keywords:** osteolysis, aseptic loosening, genomics, gene expression, inflammation, osteoclastogenesis

## Abstract

Aseptic loosening (AL) due to osteolysis is the primary cause of joint prosthesis failure. Currently, a second surgery is still the only available treatment for AL, with its associated drawbacks. The present review aims at identifying genes whose expression is altered in osteolysis, and that could be the target of new pharmacological treatments, with the goal of replacing surgery. This review also aims at identifying the molecular pathways altered by different wear particles. We reviewed preclinical and clinical studies from 2010 to 2016, analyzing gene expression of tissues or cells affected by osteolysis. A total of 32 in vitro, 16 in vivo and six clinical studies were included. These studies revealed that genes belonging to both inflammation and osteoclastogenesis pathways are mainly involved in osteolysis. More precisely, an increase in genes encoding for the following factors were observed: Interleukins 6 and 1β (IL16 and β), Tumor Necrosis Factor α (TNFα), nuclear factor kappa-light-chain-enhancer of activated B cells (NFκB), Nuclear factor of activated T-cells, cytoplasmic 1 (NFATC1), Cathepsin K (CATK) and Tartrate-resistant acid phosphatase (TRAP). Titanium (Ti) and Polyethylene (PE) were the most studied particles, showing that Ti up-regulated inflammation and osteoclastogenesis related genes, while PE up-regulated primarily osteoclastogenesis related genes.

## 1. Introduction

Hip or knee total joint replacement (THR or TKR) is the most employed surgical treatment for the final stages of joint pathologies, such as osteoarthritis (OA), arthritis, severe trauma and fractures. This procedure leads to an improvement of pain and joint functionality, and the number of THR and TKR amounts to 1.5 million every year [1,2,3]. Given the increase of the life span and of implants in young active patients, this number is expected to increase even further in the coming years (nearly 5% per year) [3,4] and, in 2030, THR will have increased by 154% worldwide [5]. Although total joint replacement (TJR) still represents the most employed and the most successful surgical orthopedic practice, it can fail despite the advances in surgical techniques and materials used for the prostheses. The main cause limiting the survival of a prosthesis is the osteolysis phenomenon, with consequent severe joint pain and mechanical instability [6]. Periprosthetic osteolysis, with an incidence ranging from 5% to 20% per year [7], is caused by many factors. The most common is the formation of wear debris on the surface of a prosthesis between its forming material and bone, leading to aseptic loosening (AL) [8]. The osteolysis phenomenon was already known during the 1970s to 1990s. After in vitro and histological studies, it was identified a fibrous capsule around artificial joints with inflammatory cells [9] and the release of pro-inflammatory mediators, such as prostaglandin E2 (PGE_2_), Interleukin 1 (IL1), tumor necrosis factor α (TNFα) and IL6 [10]. In addition, the factors that induced osteoclastogenesis in periprosthetic tissues, leading ultimately to implant loosening, were also identified [11,12].

In AL, the response of the surrounding tissue cells is the principal responsible of the implant failure [13]. More in detail, innate immune response is stimulated in the interface between bone and implant [14], and the first cells that come into play in this process are circulating peripheral blood monocytes (PBMCs), macrophages, foreign body giant cells, fibroblasts (FBs), T lymphocytes and synovial cells [14]. Consequently, local inflammation around the implant activates the production of several pro-inflammatory cytokines and chemokines, such as TNFα, IL6, IL1, PGE_2_, monocyte chemottractant protein (MCP-1) and Metalloproteinases (MMPs) [15,16]. These secreted factors can, directly or indirectly, induce chronic inflammation, tissue fibrosis, tissue necrosis and, ultimately, osteoclasts (OCs) activation, responsible for bone resorption and the osteolysis process around the implants [17,18,19]. Osteoblasts (OBs) and osteoprogenitor cells, such as bone marrow mesenchymal stem cells (BMSCs), responsible for bone formation, are also altered. Indeed, these cells, the main responsible of implant osseointegration, show a perturbed differentiation and activity, leading to a compromised bone formation activity [2,20]. Thus, both bone compartment and immune system interact in osteolysis and both bone resorption and bone formation units are compromised [20].

Besides the biological response, osteolysis is also caused by an excessive and repetitive mechanical stress. Micro-motion, due to weight and fluid pressure, enhances the osteolytic process, concurring to a further imbalance of bone metabolism and, ultimately, to prosthesis failure [21].

Several materials, such as metal (for example, Titanium (Ti), Cobalt (Co) and Chromium (Cr)), ceramic, polyethylene (PE) and polymethylmethacrilate (PMMA), can induce wear and, consequently, can prime the osteolysis process [22].

Nearly 20% of patients undergoes a revision surgery after 10–20 years from the primary surgery and the revision of a failed prosthesis (which, in 2030, will increase by 137%) leads to a new second surgical procedure [23]. If compared with a primary revision surgery, poor functional results are obtained with the second surgical approach due to poor bone quality and chronic inflammatory conditions associated with the loosened painful prosthesis, which negatively affect bone architecture and impair osteogenic capabilities [24].

Therefore, there is a need for new therapeutic approaches because, unfortunately, no therapeutic treatment is able to limit osteolysis, making the revision surgery inevitable. Several researches have been performed to understand the mechanism that leads to AL, but the exact mechanism behind this still remains unknown [25]. It is probably the combination of all theories that make AL a complex and multifactorial event, for which an effective treatment, alternative to revision surgery, has not yet been found [26].

For this purpose, it is important to know the genomics and molecular pathways altered and involved in periprosthetic wear mediated osteolysis. New therapies should act on both cellular and inflammatory pathways in order to arrest or delay the osteolysis process.

For this reason, this review intends to analyze preclinical and clinical studies, from 2010 to 2016, focusing on genetic pathways that are most frequently modified in the osteolytic process. In this way, the review will help to identify molecular pathways that more frequently altered and that could be a pharmacological target to limit the progress of osteolysis.

Secondly, this review aims at clarifying whether and what molecular pathways are modified by the different types of debris particles.

The studies, collected in this review, investigated prosthesis osteolysis due to wear debris.

Two separate search strategies were adopted to identify the articles included in this review: www.pubmed.com and www.webofknowledge.com. The limits were the language (the accepted articles were only in English language) and the year of publication (from 2010 to 2016). The search strategy, performed in this review, comprises seven years (from 2010 to 2016). However, it has allowed finding the most recent articles performed with new methodologies and new technological advancements in laboratory analysis.

With the first search strategy, the following Meshes were adopted: (“Gene Expression Regulation” [Mesh] OR “Gene Expression Profiling” [Mesh] OR “Gene Expression” [Mesh]) AND (“Osteolysis” [Mesh]) and 94 articles met these requirements.

With the second search strategy, the key words were: (Gene Expression Regulation OR Gene Expression Profiling OR Gene Expression) AND Osteolysis and 220 articles were found.

With these two searches, a total of 314 articles were red and screened.

**Figure 1:**
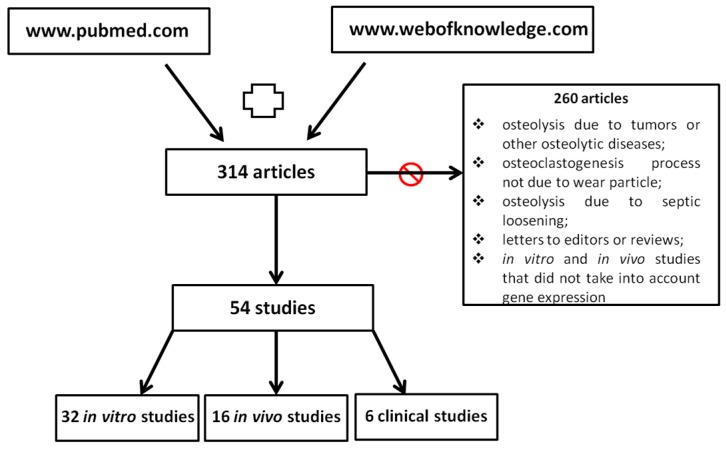
Search strategy of the review.

There were 260 excluded articles because they regarded: (1) osteolysis due to tumors or other osteolytic diseases (such as rickets, dialysis patients, arthritis and hormonal abnormalities); (2) osteoclastogenesis process not due to wear particle; (3) osteolysis due to septic loosening; and (4) letters to editors or reviews. Only the studies that compared healthy and wear-mediated osteolytic conditions, from a molecular point of view, were analyzed (Figure 1).

## 2. Gene Expression in Osteolysis

A total of 54 articles were included in this review. The studies were categorized as in vitro, in vivo and clinical studies, accounting for 32, 16 and 6 studies, respectively (Figure 1).

**Figure 2:**
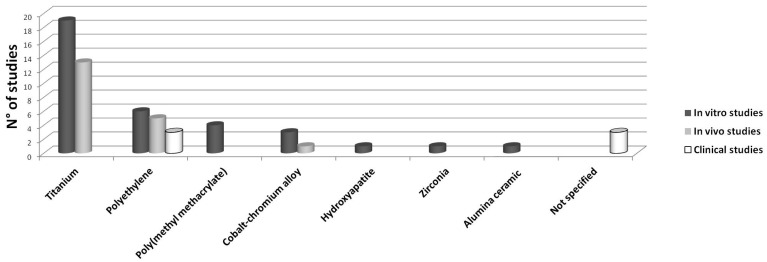
In vitro, in vivo and clinical studies dealing with different types of wear particles. The abscissa axis indicates the type of particles, while the ordinate axis indicates the number of studies dealing with each type of particles.

Each of the following paragraph reviews in vitro, in vivo or clinical studies, separately, and is divided into subparagraphs evaluating the different wear particles employed (Figure 2).

### 2.1. In Vitro Studies

As summarized in Table 1, the 32 in vitro studies included in this review were performed using different cell lines and different types of wear debris, added to the culture medium to recreate an in vitro model of osteolysis.

Most of the studies employed human or mouse cell lines. Mouse macrophage cell lines (RAW264.7) were the most used [4,6,27,28,29,30,31,32,33,34,35,36,37], followed by OB precursors derived from mouse calvaria (MC3T3-E1) [27,28,38,39,40,41] and human (Saos-2 and MG-63) [1,42,43] or rat (ROS 17/2.8) [44] osteosarcoma and monocitic (THP-1) [20,45,46] cell lines. In addition, other studies also used primary cells: mouse [28,47] or human [42,48] OBs, mouse or rat bone-marrow-derived macrophages (BMM) [28,49,50,51], human MSCs [39,52], mouse [47] or human [53] FBs and mouse peritoneal macrophages [54]. In one study, mouse calvaria [4] were cultured in toto. Regarding the different wear particles used, Ti [4,6,27,28,29,30,31,32,33,38,39,42,45,50,52,53] was the most employed, followed by PE [20,34,37,43,47,48], PMMA [36,41,51] and Co-Cr alloy [1,40,46]. Three studies compared Ti effects with PMMA [49], zirconia (ZrO_2_) [54] and aluminia ceramic (CE) [34] and one compared PE and hydroxyapatite (HA) particles [44]. Particles also had different size ranges: 6 nm–10 µm for Ti, 1.7–10 µm for PE, 0.1–10 µm for PMMA, 0.2–5.7 µm for Co-Cr alloys, 10 µm for HA, 0.2–2 µm for CE and 5 µm for ZrO_2_. Finally, the amount of particles, calculated in mg/mL, was also variable: 0.05–100 for Ti, 0.5–10 for PE, 0.1–5 for PMMA, 0.3–2.5 for Co-Cr alloys, and 1 for ZrO_2_.

#### 2.1.1. Ti Particles (19/34 Studies)

In cells of macrophagic lineage, besides an increase in the genes related to inflammation markers (such as IL6, IL1β, TNFα and Cyclooxygenase 2 (COX_2_)) and cannabinoid receptor type 2 (CB2), an increase in genes of osteoclastogenesis markers, encoding for Tartrate-resistant acid phosphatase (TRAP), Nuclear Factor Of Activated T-Cells 1 (NFATC1), Cathepsin K and Receptor Activator of Nuclear Factor κ B (RANK) was also shown. These changes were accompanied by an increase in Nitric Oxide Synthase 2 (NOS_2_), nuclear factor kappa-light-chain-enhancer of activated B cells (NFκB) and MMP9 gene expression and a reduction in genes linked to superoxide dismutase pathways [4,27,28,29,30,33,50]. One study showed an increase in TNFα, C-X-C motif chemokine 10 (CXCL10), C–C chemokine receptor type 7 (CCR7) and IL10 gene expression with microparticles of Ti in THP-1 cells in a dose-dependent manner. No effects were observed for nanoparticles [45]. One study performed with nano-sized Ti particles induced a reduction in TNFα, macrophage inflammatory protein 1-alpha (MIP-1α), MCP-1, Vascular endothelial growth factor alpha (VEGFα) and Platelet derived growth factor (PDGF) gene expression in RAW264.7 cells [6].

In cells of the osteoblastic lineage, Ti particles induced an increase in the pro-inflammatory genes and a decrease in Osteoprotegerin (OPG) gene observed in macrophagic cells and in CB2 [27,28,38,39,42]. In addition, one study analyzed osteogenesis and apoptotic pathways with microarray in hMSCs, showing an up-regulation of genes of pro-apoptotic proteins (Bcl-2-associated death promoter (BAD), Cluster of Differentiation 70 (CD70), B-cell lymphoma 2 (BCL2) and a down-regulation of anti-apoptotic and osteogenesis genes, encoding for Cartilage oligomeric matrix protein (COMP), Fibroblast Growth Factor Receptor 2 (FGFR2), Insulin-like growth factor 1 (IGF-1), Bone morphogenetic protein 6 (BMP6), Collagens (COLLs), (sex determining region Y)-box 9 (SOX9) and OPG [52].

The three studies that compared the effects of Ti with PMMA [49], ZrO_2_ [54] or CE [34] concluded that PMMA and CE behaved in the same way as Ti [34,49], while ZrO_2_ induced a more consistent reduction in inflammatory genes encoding for such as NFκB, TNFα, ILβ, IL6 and Toll-like receptor (TLRs) compared to Ti alloy particles [54]. In addition, two other studies employed Ti alloy particles, concluding that Ti alloy particles increased the expression of TNFα protein, in RAW264.7 cells [32], or of Receptor activator of nuclear factor kappa-B ligand (RANKL), in human synovial fibroblasts (SFs) in a dose-dependent manner [53].

Finally, Zhao et al. cultured mice calvaria with conditioned medium of RAW264.7 cells previously stimulated for three days with Ti particles, observing an increase in genes encoding for proteins related to osteoclastogenesis (TRAP and Cathepsin K), as well as those for Calcitonin Receptor (CALCR) and NFκB [4].

#### 2.1.2. PE Particles (7/34 Studies)

Two studies, which added ultra molecular weight polyethylene (UHMWPE) particles to RAW264.7 cells, showed an increased expression in genes encoding for inflammation and osteoclastogenesis (such as MMP9, Cathepsin K and CALCR), with more evident effects when co-cultured with DC2.4 cells [35]. In the second study, Sartori et al., comparing two concentrations of UHMWPE (0.5 and 0.75 mg/mL), showed that both concentrations reduced DNA quantification and increased RANK expression, with greater effects with 0.75 mg/mL [37].

Three further studies evaluated two different concentrations of UHMWPE particles (1:100 and 1:500), two in OBs [43,48] and one in THP-1 cell line [20]. In OBs, the authors observed that both concentrations up-regulated the gene encoding for RANKL, in a dose-dependent manner [43,48]. In one of these two studies, the higher concentration was also more effective in reducing the gene of OPG [43]. Similarly, Jablonski et al. showed an increase in the gene of *TNFα* at both concentrations, but more evident with the 1:500 dose [20].

One study compared the effects of two particle types, showing that UHMWPE and HA, respectively, reduced and increased OPG gene expression [44].

Finally, high density polyethylene (HDP) particles increased the expression of gene for IL6 in primary FBs harvested from mice subcutaneous tissues of cranial capsules and in OBs [47].

#### 2.1.3. PMMA Particles (3/34 Studies)

Stimulated RAW264.7 cells increased genes related to osteoclastogenesis markers (TRAP, Cathepsin K and RANK) [36].

In BMM and OB precursors, PMMA particles increased the expression of genes related to inflammation and immune response (such as those encoding for IL1β, C–C chemokine receptor type 2-CCR2 and major histocompatibility complex 2-MHCII) while decreasing those concerning OB differentiation, such as Runt Related Transcription Factor 2 (RUNX2), Osterix (OSX) and Osteocalcin (OCN) [41,51].

#### 2.1.4. Co-Cr Alloy (3/34 Studies)

Three studies compared the effects of Co-Cr alloys and their ion forms Co (II) and Cr (III) [41], Co-Cr-Molybdenum (Mo) alloy with and without Nickel (Ni) [1] or different Co-Cr-Mo alloy concentrations (1:10, 1:100, 1:200, 1:500, and 1:1000) [46].

In the first study, Co-Cr alloys and ions reduced the expression of OB differentiation markers (OPG, RUNX2 and OCN), while Co-Cr alloys and Co (II) also increased genes related to inflammation and osteoclastogenesis (TNFα, IL6, RANKL, CCL2 and NFATC1): this effect was more evident with Co-Cr alloy [40]. Co-Cr-Mo alloys with and without Ni increased the expression of gene for TNFα and the presence of Ni reduced their expression in OBs [1].

In the last study, the expression of the gene encoding for IL8 increased in a dose-dependent manner with the addition of Co-Cr-Mo alloy in the medium of monocitic cells at a concentration greater than 1:500 [46].

#### 2.1.5. Other Than Genes

Besides gene pathways changes, it is fair to point out that all particle types in all treated cells increased cell apoptosis, membrane damages and OC number and activity. In addition, an increase in the production of reactive oxygen species (ROS), p38 mitogen-activated protein kinases (p38 MAPK) phosphorylation, nuclear accumulation and phosphorylation of p65, NFκB and Lactate dehydrogenase (LDH) enzyme were observed. In cells of the osteoblastic lineage, wear particles reduced OPG, Alkaline phosphatase (ALP), Collagen I (COLL I) and OCN production; cell viability; and mineralization.

### 2.2. In Vivo Studies

All 16 in vivo studies were performed in mice, except for one carried out in rats [55]. Three different models of osteolysis were adopted: air-pouch [16,29,33,56,57,58,59,60], calvarial [18,53,61,62,63,64] and pin-implantation [65] models. One study compared two methods to induce osteolysis in vivo: fluid pressure and particle injection [55].

Opposite to what was observed in vitro, these in vivo studies used only two types of wear particles: Ti [16,29,33,53,55,56,57,58,59,60,64,65], the most employed ones, and PE [18,61,62,63]. The size of Ti particles ranged between 0.1 and 20 µm while PE particles were 7 [61,62,63] or 5.1 [18] µm. The amount of Ti particles varied from 10 to 150 mg/mL in the murine air-pouch model [16,29,33,53,56,57,58,59,60,65] to 300 mg/mL in the murine calvarial model [64]. The amount of PE was between 0.5 and 20 mg/mL [18,61,62,63] (Table 2).

#### 2.2.1. Ti Particles (12/16 Studies)

In the most numerous group of in vivo studies, which employed the murine air-pouch model, the authors agreed that Ti particles increased the expression of genes encoding for inflammatory and catabolic proteins (such as MMP9, TNFα, COX_2_, IL1β and TNF receptor associated factor 6 (TRAF6)), for proteins related to osteoclastogenesis (such as RANK, RANKL and NFATC1), for growth factors (such as VEGFA) and CB2 [16,29,33,58,59,60]. These results were also confirmed in the study employing metallic wear particles obtained from the stem of hip prosthesis containing not only Ti, but also Co and Cr [56].

Two studies were performed in a murine calvarial model and the authors observed a reduction in genes encoding for OPG and an increase in those for RANKL [53,64].

Likewise, in the only in vivo study using the pin-implantation model, Jiang et al. found that intra-articular injection of Ti-challenged OBs or Ti alloy particles itself, increased the expression of genes linked to inflammation and osteoclastogenesis (MMP2, IL1β, TNFβ, RANKL and TRAP), more evident with Ti-challenged OBs than with Ti [65]. Similar results were also observed in another study that employed Ti alloy particles instead of pure Ti, in which the expression of genes, encoding for MMP9, TNFα, RANK and RANKL, increased after Ti alloy particles injection [57].

Finally, Nilsson et al. compared two methods to induce osteolysis in rats, by fluid pressure on Ti particles injections, observing that both models induced an increase in inflammation and osteoclastogenesis, even if the injection method was more effective [55].

#### 2.2.2. PE Particles (4/16 Studies)

All studies used PE particles injections in murine calvarial model [18,61,62,63].

An increase in genes of inflammation and osteoclastogenesis, Cathepsin K, TNFα, IL6, IL1β, NFκB, COX_2_, RANK and RANKL was observed [18,61,62,63]. In one study, this was dose-dependent [62]. Among these studies, one observed the same results also in ovariectomized (OVX) rats treated with estrogen; however, in OVX rats not treated with estrogen, PE particles did not induce significant differences [18].

#### 2.2.3. Other Than Genes

As previously mentioned for in vitro studies, protein production and structural changes were also observed in in vivo studies.

At histological level, the following changes were observed: increase in OCs number, bone resorption, pouch membrane thickness and inflammatory cell infiltrate, bone porosity, osteonecrosis and osteolytic area. In addition, Ti and PE particles reduced bone mineral density (BMD), bone volume (BV), Bone volume/Tissue volume (BV/TV), trabecular thickness (Tb.Th) and pulling force.

As for protein production, an increased phosphorylation of p-38 MAPK was observed.

### 2.3. Clinical Studies

As outlined in Table 3, six studies analyzed human tissues and cells from patients affected by periprosthetic osteolysis and undergoing THR or TKR [19,66,67,68,69,70]. More precisely, synovial and pseudosynovial membranes [19,67,68], interfacial membranes [67], joint capsules [70], peripheral blood [66] and hPBMCs [69] were the retrieved tissues and cells.

Among these six studies, three did not specify the materials of the prosthesis [19,67,68] and threee dealt with osteolysis due to prosthesis based on PE [66,69,70].

In interfacial membranes, harvested from patients affected by osteolysis of hip implants, Pan et al. showed an increased expression of the gene of Macrophage Migration Inhibitory Factor (MIF) compared to synovial membranes harvested from hip joints of fractured patients, considered as healthy control [67]. Tomankova et al. also investigated the differences in gene expression of pseudosynovial membranes between hip and knee osteolytic implants compared to pseudosynovial membranes of patients affected by hip and knee OA. When compared to knee OA, TKA pseudosynovial tissue showed an increase of alternative macrophage activation marker (Chitinase 1-CHIT1), chemokine (IL-8) and MMP9 and a decrease of TNFα, OPG and BMP4 genes. Analyses between TKA and THA failed groups revealed lower Chemokine (C–C motif) ligand 3 (CCL3) and dendritic cell-specific transmembrane protein (DC-STAMP) mRNA expression in TKA [68].

Synovial membranes of failed TKA patients exhibited higher expression of the gene encoding for TNF related weak inducer of apoptosis (TWEAK), a pro-apoptotic protein, in comparison to those harvested from healthy or osteoarthritic patients [19].

Peripheral whole blood samples from subjects affected by OA and implanted with THA were harvested and genotyped by MacInnes et al. Half of the patients with osteolysis had eight single nucleotide polymorphisms associated with susceptibility to osteolysis: four of these lie within bone resorption (RANK), and the other four within Kringle containing transmembrane protein 2 (KREMEN2), OPG, Secreted frizzled related protein 1 (SFRP1) and the regulator of inflammatory signaling (TIR domain containing adaptor protein (TIRAP)), respectively. None of the polymorphisms was associated with time to failure [66]. Saad et al. analyzed tissue samples from patients undergoing revision surgeries for osteolysis and synovial samples from osteoarthritic patients undergoing primary surgeries. They observed that genes encoding for Osteoclast-associated immunoglobulin-like receptor (OSCAR), Fc receptor common γ subunit (FcRg), Triggering receptor expressed on myeloid cells 2 (TREM2) and TYRO protein tyrosine kinase binding protein (DAP12) were more expressed by the large multi-nucleated cells in osteolysis samples, compared with little or no expression in synovial tissues obtained by osteoarthritic synovial membranes [70]. Finally, hPBMCs were harvested from patients affected by osteolysis due to failed hip/knee prosthesis and from patients who underwent primary hip/knee prosthesis due to OA. Gordon et al. observed that the expression of genes for IL1α, IL1β, IL6, IL10, IL18 and TNFα were reduced in the first group of patients [69].

### 2.4. Other Than Genes

Besides the changes in gene expression, an increase in the number of OCs, macrophages, FBs, monocytes, histiocytes, giant cells and in the percentage of resorbed area was also observed in these studies.

## 3. Discussion

The primary cause of failure of a long-term prosthetic survival is AL due to osteolysis. The biological (the responses of the host tissues to the wear prosthesis debris) and mechanical aspects are the main factors that trigger this process [71]. Unfortunately, the only currently available therapeutic treatment for AL is the surgical revision with its associated drawbacks [23,24]. Therefore, nonsurgical therapeutic interventions are desirable and the preclinical research is intended for this purpose.

The present review aims at identifying genes whose expression is modified in the osteolysis process, by collecting and analyzing recent preclinical and clinical literature studies between 2010 and 2016. Even if the included studies analyzed different aspects of osteolysis, such as proteins production and periprosthetic bone architecture, the review and its discussion focus prevalently on the genomics of osteolysis. The identification of the molecular patterns that are mainly modified in osteolysis, in comparison to a healthy condition, should help the development of nonsurgical strategies that can act directly on the genes involved in osteolysis.

The first evidence that emerges from this review is that both inflammation and osteoclastogenesis processes are the main factors involved in osteolysis, resulting in bone loss. These two processes are not entirely separate, but they are interacting, also because some genes belong to both.

In vitro studies, which are the most numerous, tend to recreate a microenvironment similar to that observed in vivo in presence of osteolysis, by adding wear particles to cell culture medium. These studies take into consideration several cell types of the monocyte/macrophage and osteoblast (mature OBs and OB precursors) lineages and found that genes encoding for pro-inflammatory factors, especially IL6, TNFα and IL1β, are mainly increased. These genes also induce OC formation and reduce OB differentiation, leading to bone loss, periprosthetic osteolysis and, consequently, implant failure. *Nfκb* gene, the key factor that regulates the inflammatory processes located upstream of pro-inflammatory genes, is also increased [72]. A lower number of in vitro studies also evidenced an up-regulation of oxidative stress with increased expression of genes for NOS_2_ and COX_2_ after wear particle stimulation, while the up-regulation of the anti-inflammatory cytokine IL10 seemed to be linked to a protective effect of macrophages against inflammation [29].

The second group of genes, whose expression is increased in osteolysis, belongs to the osteoclastogenesis process and encodes for Cathepsin K, NFATC1 and TRAP. More precisely, NFATC1 is the master regulator of osteoclastogenesis and OC function, and regulates the expression of genes for Cathepsin K and TRAP, involved in OC differentiation. It also seems to be involved in the inflammatory process because it is activated by TNFα [73]. To a lesser extent other two important genes, MMP9 and MCP-1, are also increased. The first is an osteoclastogenesis related gene, because concurs to OC activation and maturation and is activated by TNFα and interleukins; the second is an important chemokine useful for the recruitment of monocytes and macrophages in sites near the prosthesis, producing inflammation and osteoclastogenesis [60,74].

OPG/RANK/RANKL axis is also affected by osteolysis and an increase in RANK and RANKL and a decrease in gene of OPG were observed after wear particles exposure. RANKL, up-regulated by TNFα, is the controller of osteoclastogenesis by binding to its receptor RANK on OCs. On the other hand, OPG antagonizes RANKL binding to RANK, thus inhibiting OC differentiation [75].

Finally, few authors evaluated the group of genes expressed by OBs or osteoprogenitor cells. Genes encoding for RUNX2, the master regulator of OB proteins, for example Osterix and OCN, concurring to OB maturation, are down-regulated in osteolysis [2].

Despite in vivo studies are less numerous than in vitro studies, the same pattern of gene regulation emerged also in these studies. Most of the in vivo studies adopted the murine air-pouch [16,29,33,56,57,58,59,60] and calvarial [18,53,61,62,63,64] models. The first is classically used because it can recreate inflammatory components similar to those of the periprosthetic tissues of osteolytic patients [76]. It is useful for the evaluation of inflammatory and resorption aspects of osteolysis, even if it does not reproduce the physiological situation [77]. The second is a quick low cost osteolysis model that represents a more physiological model because the evaluations are performed in a bone microenvironment. However, it disregards the biomechanics aspects and lacks endochondral ossification evaluations [34,78]. The gold standard model to evaluate osteolysis in animals is the pin implantation, because it resembles the clinical situation more closely [79]; however, this is employed only by one study in this review [65].

While in vitro most of the modified genes are related to inflammation, in vivo they are linked to osteoclastogenesis. The up-regulation of RANKL and RANK genes seems to be more evident in vivo than in vitro and, to a lesser extent, also NFATC1, TRAP and Cathepsin K were increased. Similar to the in vitro studies, OPG gene expression was also reduced in vivo.

As for inflammation, genes encoding for TNFα and IL1β are the most altered, followed by MMP9, COX_2_, NOS_2_ and IL6.

One gene whose expression is up-regulated in both in vitro and in vivo studies is CB2, the cannabinoid receptor that regulates bone mass, highly expressed also in inflammatory tissues and known to induce osteoclastogenesis [80].

The second important aspect to take into consideration regards the genetic pathways modified by different wear particles. Ti particles are the most used in preclinical studies followed by PE, but no uniformity in particle sizes and quantity is found among the studies. In addition, different particle types alter different gene pathways. In vitro, besides Ti and PE, also PMMA and Co-Cr alloys were used, while in vivo, Ti and PE are the only wear particles employed. In vitro, Ti affects the inflammation process, mostly modifying gene for TNFα, while PE and PMMA increase osteoclastogenesis, the first up-regulating RANKL and the second Cathepsin K and TRAP. Co-Cr alloys reduce osteoblastogenesis (genes for OPG, RUNX2 and Osterix). In vivo, there is consensus that both Ti and PE particles are more effective on osteoclastogenesis, increasing RANKL gene expression.

Despite the great variability found in particle size, most Ti and PE particles used in vitro had diameters of 4–4.5 µm [31,33,38,42,49] and 1.7 µm [20,43,48], respectively, with dimensions never exceeding 10 µm. In regards to in vivo models, Ti ranged from 0.1 to 20 µm [55,57,59,60], while PE particles dimensions were usually about 7 µm [61,62,63].

In vitro and in vivo studies employed Ti as Ti alloys or pure Ti particles, but Ti alloys were used by fewer studies than pure Ti. Anyway, Ti alloys and pure Ti influenced the expression of the same genes, encoding for inflammation and osteoclastogenesis pathways.

No conclusion can be drawn about the concentrations used in vitro and in vivo, because of the great variability among the studies. In this regard it is important to underline that some authors compared different particles dimensions and different wear particle concentrations. Even if only one study observed a reduction in gene for OPG with Ti microparticles and not with nanoparticles [45], the increase of osteoclastogenesis and the decrease of osteoblastogenesis gene expression were dose-dependent in some studies [20,37,43,45,46,48,53]. Moreover, some authors also compared different types of particles in vitro. Although PMMA and CE particles behave similarly to Ti [34,49], ZrO_2_ reduced pro-inflammatory genes more than Ti [54]. The addition of Ni to Co-Cr-Mo alloys reduced gene for TNFα [1] and Co-Cr alloy increased osteoclastogenesis to a greater extent than the ion form [40].

On the other hand, only one study compared different concentrations of PE particles in vivo, observing an increase in genes encoding for NFκB, TNFα, IL1β, RANKL and COX_2_ in a dose-dependent manner [62].

A separate discussion should be made for clinical studies, the smallest number of studies of this review, which used human tissues and cells taken from patients affected by periprosthetic osteolysis. These studies used tissues and cells harvested from osteoarthritic patients as control group, given the difficulty in finding the material from healthy subjects. Half of the studies did not specify the material of which the prosthesis is made of [19,67,68] and in the other half the prostheses used were in PE [66,69,70]. Analyzing these studies, new genes emerged, different from those evaluated in the previous preclinical studies. An increase in the genes encoding for MIF was observed in osteolytic tissues, a protein considered a regulator of inflammation and activator of the expression of pro-inflammatory cytokines (such as TNFα, IL6 and MMPs) [81]. The other genes encode for activators of macrophages, pro-apoptotic factors and inducers of OCs (such as OSCAR, TREM2) or factors linked to in innate immune responses (such as DAP12 and FcRg) [82,83,84]. In addition, genes associated to OB development (such as OPG and BMP4) are reduced. No study analyzed the same genes.

A limitation of the studies included in this review is principally the difficulty in finding a standardization regarding the amount and the size of the particles to be used in preclinical studies. In the author’s opinion, in vitro and in vivo studies analyzing osteolysis should adopt particles dimensions and concentrations of those found in periprosthetic tissue of AL patients, and thus clinically relevant. The periprosthetic debris sizes found in clinical practice after prosthesis implantation are nearly 99 nm for Ti and between 0.1 and 0.5 µm for UHMWPE for hip and 280 µm for knee [85,86]. In addition, genome-wide expression studies are still missing, so it may be worthwhile to perform systematic analyses in future studies.

The search strategy performed in this review comprises seven years (from 2010 to 2016). However, it has allowed finding the most recent articles on the issue in two separate databases.

## 4. Conclusions

To summarize the main findings of the review (Table 4): (1) osteolysis mainly affects genes belonging to inflammation and osteoclastogenesis pathways, followed by those of osteoblastogenesis; (2) frequently up-regulated inflammation and osteoclastogenesis genes are those encoding for IL6, TNFα, IL1β and NFκB, for inflammation, and NFATC1, Cathepsin K and TRAP for osteoclastogenesis; (3) down-regulated genes of osteoblastogenesis are RUNX2, OCN and Osterix; (4) no uniformity exists in the different models employed in vitro and in vivo, because different wear particle types, dimensions and amounts are employed in different studies; (5) Ti and PE, especially UHMWPE, are the most evaluated particles in preclinical studies; (6) in vitro and in vivo studies agree that Ti particles up-regulate genes related to inflammation and osteoclastogenesis and PE those of osteoclastogenesis; and (7) a link between immune cell activation, local inflammation and OC recruitment is observed.

This review is the first that collects preclinical and clinical studies about in vitro, in vivo and clinical models of osteolysis to identify the main genetic pathways altered in osteolysis. Further studies that analyzed human tissues and cells are necessary in order to draw a more accurate conclusion on osteolysis genes involved in human tissues of patients affected by osteolysis.

## Figures and Tables

**Table 1:** In vitro studies.

Type of Wear Particles	Cell Type	Evaluations	Results: Osteolysis vs. Control	Ref.
100 mg/mL Ti alloy particles (ø < 8 µm)	Raw 264.7	Gene expression (*Tnf*) Protein levels (TNFα, p-TAK1, TAK1, p-P38, p-38, cytosolic and nuclear p50 and p65)	↑ TAK1, *Tnf*, TNFα, nuclear and cytosolic p50, nuclear p65, p-38	[32]
1 mg/mL ZrO_2_ or Ti alloy particles (ø 5 µm)	Mice peritoneal macrophages	Gene expression (*Tlr2, Tlr3, Tlr4, Tlr9, Myd88, Ticam2, Tnfrsf11a, Tnf, Il1, Il6*) Protein levels (IL1β, IL6, TNFα)	↑ *Tlr2, Tlr3, Tlr4, Tlr9, MyD88, Ticam2,* *Nfκb*, *Tnf*, *Il1, Il6*, TNFα. ↑ *Tlr2, Tlr9;* *↓ Tlr3, Tlr4, MyD88, Ticam2, Nfκb, Tnf, Il1, Il6* in ZrO_2_ than Ti	[54]
0.05, 0.5, 1 mg/mL Ti alloy particles (ø 0.52 µm)	SFs from OA pz	Protein levels (IRE1-α, CHOP, RANKL, OPG, sRANKL) Gene expression (*TNFSF11, TNFRSF11B*) OCs number	↑ CHOP, *TNFSF11*, RANKL, sRANKL, IRE1α (dose-dependent manner)	[53]
0.1 mg/mL Ti particles (ø < 10 µm)	RAW264.7	Cell viability Gene expression (*Tnf*, *Il1*, *Il6*, *Il10*, *Ccl2*, *Ccl3*, *Nos_2_*, *Cox_2_*) Protein levels (TNFα, IL1β, IL6, IL10, MCP-1, MIP-1α, iNOS, COX_2_)	↑ *Tnf*, *Il1*, *Il6*, *Il10*, *Ccl2*, *Ccl3*, *Nos_2_*, *Cox_2_*, TNFα, IL1β, IL6, MCP1, MIP1α, iNOS, COX_2_ ↓ IL10	[29]
0.1 mg/mL Ti particles (ø 2.6 mm)	Raw264.7	NO production Intracellular ROS Lipid peroxidation Gene expression (*Nos_2_*, *Nox_1_*, *Nox_2_*, *Sod1*, *Nos_3_*, *Gpx*, *Gr*, *Catk*, *Tnf*, *Nfκb*) SOD, CAT, GR activities Protein levels (TNFα, p-NFκB -p65, NFκB-p65)	↑ NO, ROS, N*os_2_*, *Nox_1_*, *Nox_2_*, *Tnf*, *Nfκb*, TNFα, p- NFκB -p65, GR, SOD, CAT ↓ *Sod1*, *Nos_3_*, *Gpx*	[30]
0.1 mg/mL Ti particles (ø 4.5 µm)	RAW264.7	CB2 activity Bone resorption pit assay OCs number Protein levels (IL1β, TNFα) Gene expression (*Cnr2*, *Tnfrsf11a*, *Ctsk*)	↑ OCs number, *Cnr2*, *Tnfrsf11a*, *Ctsk*, TNFα, IL1β, CB2 activity, resorption area	[31]
0.1 mg/mL Ti particles (ø 4.50 µm)	RAW264.7	Protein levels (IL1β, TNFα) Gene expression (*Il1*, *Tnf*)	↑ *Il1*, *Tnf*, IL1β, TNFα	[33]
Calvaria with CM of RAW264.7 stimulated with 1 mg/mL Ti particles RAW264.7 with 1% Ti particles (ø < 3.6 µm)	Calvaria from mice (5-day old) and RAW264.7	OCs number Gene expression (*Trap*, *Cstk*, *Calcr*, *Nfκb*, *Tnf*, *Il1*, *Il6*, *Cox_2_*, *Nos_2_*) Protein levels (TNFα, IL1β, IL6, cytosol and nuclear p65)	Calvaria: ↑ OCs number, *Trap*, *Cstk*, *Calcr*, *Nfκb*. RAW264.7: ↑ *Tnf*, *Il1*, *Il6*, *Cox_2_*, *Nos_2_*, NOS_2_, IL1β, IL6, TNFα, nucleus p65; ↓ cytosol p65	[4]
4x10^4–^4x10^6^ particles/mL TiO_2_-N (ø <0.1 µm) or TiO_2_-M (ø <5 µm)	THP-1	Protein levels (TNFα, IL-10) Gene expression (*TNF*, *IL10*, *CXCL10*, *CCR7*, *MRC1*)	TiO_2_-M: ↑ TNFα, IL-10, *TNF*, *IL10*, *CXCL10*, *CCR7* (at the highest concentration)	[45]
0.1 mg/mL Ti particles (ø 3.6 µm)	Mice BMM	OCs number Bone resorption assay Gene expression (*Ctsk*, *Trap*, *Nfatc1*, *Mmp9*, *Il1*, *Il6*, *Tnf*) Protein levels (TNFα, IL1β, IL6)	↑ OCs number, *Trap*, *Nfatc1*, *Ctsk*, *Mmp9*, *Tnf*, *Il1*, *Il6*, TNFα, IL1β, IL6, resorbed area	[50]
TiO_2_ (ø 8 nm)	RAW264.7	Cell viability Gene expression (*Tnf*, *Il1*, *Cxcl2*, *Ccl2*, *Vegfa*, *Pdgfa*)	↓ cell viability, *Cxcl2*, ↑ *Ccl2*	[6]
2.5 mg/mL Ti particles (ø 4.5 µm)	MC3T3-E1	Gene expression (*Cnr2*, *Tnfrsf11b*, *Tnfsf11*) Caspase-3 activity Protein level (CB2, ALP, OCN, COLL I); Cell viability Apoptosis Mineralization	↑ *Cnr2*, CB2, apoptosis, caspase-3 activity, *Tnfsf11* ↓ cell viability, ALP, COLL I, OCN, mineralization, *Tnfrsf11b/Tnfsf11*	[38]
0.05% (*v*/*v*) Ti particles (ø 0.3–1 µm)	MC3T3-E1, RAW264.7, mice primary OBs and BMMs	Gene expression (*Il6*, *Cox_2_*, *Il1*, *Tnf*, *Fam213A*) Protein levels (p-ERK) Cell membrane damage and death	MC3T3-E1:↑ *Il6*, *Cox_2_*, *Fam213A*, membrane damage ↓ viability. RAW264.7: ↑ *Il6*, *Cox_2_*, *Tnf*, *Il1* membrane damage ↓ viability OBs, BMMs: ↑ *Il6*, *Cox_2_*, *Fam213A*, *Il1*, *Tnf*, p-ERK	[28]
0.1 mg/mL Ti particles (ø < 10000 µm)	MC3T3-E1, RAW264.7	Gene expression (*Tnf*, *Il6*, *Il1*, *Trap*, *Nfatc1*, *Csf1*, *Tnfsf11*, *Tnfrsf11b*) Protein levels (TNFα, NFATc1, BMP2) Cell viability OCs number; Bone pit resorption assay	RAW264.7: ↑ *Tnf*, *Il6*, *Il1*, *Nfatc1*, NFATc1, OCs number, resorbed area. MC3T3-E1: ↑ *Tnfsf11*, *Il6*	[27]
Ti particles (ø 1–3 µm)	MC3T3-E1, hMSCs	Protein levels (p-p44; p-p42, pCREBβ, IL6, PGE_2_) Gene expression (*Il6*, *Cox_2_*) OCs number	↑ *Il6*, *Cox_2_*, p-ERK, pCREBPb, IL6, PGE_2_, OCs number	[39]
2.5 mg/mL Ti particles (ø < 4 µm)	Saos-2, OBs from pz	Cell viability Protein levels (IL6) Gene expression (*Il6*)	↑ IL6, *Il6*	[40]
100 particles/cell TiO_2_ particles (ø 0.43 µm)	hMSC from OA pz	ALP activity Mineralization Cell viability Protein levels (IL6, IL8) Gene expression (*TNFRSF11B*, *BCL10*, *COMP*, *FGFR2*, *IGF1*, *BMP6*, *SOX9*, *COLLs*, *CSF2*, *BAD*, *CD70*, *BCL2*)	↓ cell viability, ALP activity, mineralization, *TNFRSF11B*, *BCL10*, *COMP*, *FGFR2*, *IGF1*, *BMP6*, *SOX9*, *COLLs*; ↑ *CSF2*, *BAD*, *CD70*, *BCL2*	[52]
0.1 mg/mL Ti (ø 4.5 µm) or PMMA particles (ø 6 µm)	Mice BMM	Phagocytosis OCs number Gene expression (*Nfatc1*, *Trap*, *Mmp9*, *CtsK*, *Calcr*) Bone pit resorption assay Protein levels (TNFα, NFATc1)	↑ OCs number, *Nfatc1*, *Trap*, *Mmp9*, *Ctsk*, *Calcr*, resorbed area, TNFα, NFATc1, phagocytosis	[49]
1:100 (cells: particles) Ti or CE particles (ø 0.2 or 1.2 µm)	RAW264.7	Gene expression (*Pik3cb*, *Pik3r2*, *Tnf*) Protein levels (TNFα, p-AKT)	↑ *Pik3cb*, *Tnf*, TNFα, p-AKT	[34]
5 mg/mL UHMWPE particles (ø 2.6 µm)	RAW264.7 alone or co-cultured with DC2.4	OCs number Protein levels (TNFα, MCP-1, p65, p-p65) Gene expression (*Mmp9*, *Calcr*, *Cstk*)	Raw264.7: ↑ OCs number, TNFα, MCP-1, p-p65, p65, *Mmp9*, *Ctsk*, *Calcr*. Co-cultures: ↑ OCs number, MCP-1, p-p65, p65, *Mmp9*, *Ctsk*, *Calcr*	[35]
0.5 or 0.75 mg/mL UHMWPE particles (ø < 9 µm)	RAW264.7	DNA quantification Live/Dead Gene expression (*Nfatc1*, *Tnfrsf11a*, *Tnfsf11*, *Tnfrsf11b*) Pit resorption assay Protein levels (RANKL, OPG, TNFα, PGE_2_) NFκB assay	↓ DNA quantification (at 3 days) ↑ *Tnfrsf11a* (at 7 days), *Tnfsf11* (at 3 days), RANKL, TNFα, PGE_2_, NFκB activation ↑ *Tnfsf11a* (at 3 days), *Nfatc1*, RANKL, resorption activity in 0.75mg/mL PE than 0.5 mg/mL	[37]
1:100 or 1:500 (cells: particles) UHMWPE particles (ø 1.74 µm)	MG-63	Gene expression (*TNFSF11*, *TNFRSF11B*) Protein levels (RANKL, OPG) ALP activity	↑ RANKL, *TNFSF11* ↓ ALP activity. ↑ *TNFSF11;* ↑ *TNFRSF11B*, ALP activity in 1:500 than 1:100	[43]
1:100 or 1:500 (cells: particles) UHMWPE particles (ø 1.74 µm)	Primary hOBs from fractured pz	Gene expression (*TNFRSF11B*, *TNFSF11*) Protein levels (RANKL, OPG) ALP activity	↑ *TNFSF11*, RANKL ↓ ALP activity ↑ *TNFSF11* in 1:500 more than 1:100	[48]
1:100 or 1:500 (cells: particles) UHMWPE particles (ø 1.74 µm)	THP-1	Gene expression (*TNFRSF11A*, *TNF*) Protein levels (RANK, TNFα, IL1β, IL6, CGRPR1)	↑ CGRPR1, TNFα, IL1β, IL6, *TNF* ↑ IL1β, TNFα, *TNF*, IL6 in 1:500 more than 1:100	[20]
1 mg/mL UHMWPE, HA particles (ø 10 µm)	ROS 17/2.8	Protein levels (TNFα, RANKL, OPG) Gene expression (*Tnfrsf11b*, *Tnfsf11*)	UHMWPE: ↑ TNFα ↓ OPG, *Tnfrsf11b.* HA: ↑ TNFα, OPG, *Tnfrsf11b*	[44]
10 mg/mL HDP particles (ø 8.59 µm)	Primary FBs from the subcutaneous tissues of cranial capsules of mice, OBs from mice	Protein levels (IL6) Gene expression (*Tnfsf11*, *Tnfrsf11b*, *Il6*, *Il1*, *Tnf*, *Cox_2_*)	FBs: ↑ IL6, *Il6*	[47]
5 mg/mL PMMA particles (ø 0.33 µm)	RAW264.7	TRAP function OCs number Gene expression (*Ctsk*, *Trap*, *Tnfrsf11a*, *Cys1)*	↑ TRAP function, OCs number, *Trap*, *Ctsk*, *Tnfrsf11a*	[36]
2 mg/mL PMMA particles (ø 0.1–10 µm)	Rat BMM	Gene expression (*Il1*, *MhcII*, *Il10*, *Tgfb1*, *Ccr2*) Protein levels (iNOS)	↑ *Il1*, *MhcII*, *Il10*, *Tgfb1*, *Ccr2*, iNOS	[51]
1 mg/mL PMMA particles (ø 6 mm)	MC3T3-E1	Cell viability LDH ALP activity; Gene expression (*Nfκb*, *Runx2*, *Sp7*, *Bglap*)	↑ LDH, *Nfκb* ↓ cell viability, ALP activity, *Runx2*, *Sp7*, *Bglap*	[41]
0.3 or 2.5 mg/mL Co-Cr alloy particles (ø 5.7 µm); 62 or 500 µm Co (II) or Cr (III) (ø < 0.2 µm)	MC3T3-E1	Cell viability ALP activity Gene expression (*Ccl2*, *Tnf*, *Il6*, *Tnfsf11*, *Tnfrsf11b*, *Nfatc1*, *Runx2*, *Sp7*, *Lrp5*)	Co-Cr alloy: ↓ cell viability, ALP activity, *Runx2*, *Sp7* ↑ *Nfatc1*, *Tnf*, *Il6*, *Ccl2* (dose-dependent manner). Co(II): ↓ cell viability, ALP activity, *Tnfrsf11b*, *Lrp5*, *Runx2*, *Sp7* ↑ *Tnfsf11*, *Tnf*, *Nfatc1* (dose-dependent manner). Co-Cr alloy: ↑ *Ccl2*, *Tnf*, *Nfatc1* than Co(II)	[40]
1 mg/mL Co-Ni-Cr-Mo-alloy or Co-Cr-Mo-alloy (ø < 5 µm)	MG-63, Saos-2	Gene expression (*CXCR4*, *TNF*) Protein levels (CXCR4)	↑ CXCR4, *CXCR4*, *TNF*. ↓ CXCR4, *CXCR4*, *TNF* in Co-Ni-Cr-Mo more than Co-Cr-Mo	[1]
1:10, 1:100, 1:200, 1:500, 1:1000 (cells: particles) Co-Cr-Mo alloy from THA femoral head (ø 0.81 µm)	THP-1	Cell viability Cytosol and nuclear protein levels (I-KB, NFκB, IL1β, TNFα, IL8) Gene expression (*Il8*)	↑ IL1β (at 1:1000), IL8, *Il8* (at ≥1:500), nuclear NFκB ↓ cytosolic I-KB, cytosolic NFκB, cell viability (at 1:1000)	[46]

**Table 2:** In vivo studies.

Type of Wear Particles	Animal Number, Species and Model	Evaluations	Results: Osteolysis vs. Control	Ref.
15 mg/mL Ti alloy particles (ø 0.1–20 µm)	30 Female BALB/c mice (8–10 weeks old). Murine air-pouch model (also with injection of bone)	Infiltrated cells Pouch membrane thickness Bone erosion OCs number Protein levels (MMP9, TNFα, RANK, RANKL) Gene expression (*Mmp9, Tnf, Tnfrsf11a, Tnfsf11*)	↑ Infiltrated cells, pouch membrane thickness, bone erosion, MMP9, TNFα, RANK, RANKL, OCs number, *Mmp9, Tnf, Tnfrsf11a, Tnfsf11*	[57]
Ti-alloy pin (ø 0.8 mm, length 5 mm)	36 BALB/c mice (10–12 weeks). Mouse pin-implantation model (before and after Ti-alloy implantation, 10 µL and 40 µL of Ti-challenged OBs and Ti particles were injected, respectively)	BMD Bone resorption Infiltrated cells Periprosthetic membrane thickness peak pulling force OCs number Gene expression (*Sp7, Nfatc1, Runx2, Ccl2, Il1, Tnfsf11, Trap, Mmp2*)	↓ Pulling force, BMD ↑ bone resorption, infiltrated cells, periprosthetic membrane thickness, OCs number, *Mmp2, Il1, Tnf, Tnfsf11, Trap*. ↑ periprosthetic membrane thickness, OCs number, *Mmp2, Il1, Tnf, Tnfsf11, Trap* in Ti-challenged OBs more than Ti	[65]
10 mg Ti particles (ø < 10 µm)	16 female BALB/C mice (6 weeks old). Murine air-pouch model (also with injection of bone)	Gene expression (*Tnfsf11*, *Tnfrsf11b*, *Vegfa*, *Traf6*) Protein levels (RANKL, OPG, VEGF, TRAF6); Pouch membrane thickness Infiltrated cells Bone erosion Apoptosis assay CD68+ cells	↑ *Tnfsf11*, *Vegfa*, *Traf6*, RANKL, VEGF, TRAF6, RANKL/OPG, CD68+ cells, apoptotic CD86+ cells, pouch membrane thickness, infiltrated cells, bone erosion	[29]
10 mg/mL Ti particles (ø < 3.6 µm)	20 female BALB/c mice (6–8 weeks old). Murine air-pouch model (also with injection of bone)	Protein levels (COX_2_, RANKL, PGE_2_, IL1β, TNFα) Gene expression (*Tnfrsf11a*, *Tnfsf11*, *Cox_2_*, *Tnf*, *Il1*) OCs number	↑ *Cox_2_*, *Tnfrsf11a*, *Tnfsf11*, *Tnf*, *Il1*, TNFα, IL1β, PGE_2_, COX_2_, RANKL, OCs number	[16]
10 mg/mL Ti particles (ø < 3.6 µm)	20 female BALB/c mice (8–10 weeks old). Murine air-pouch model (also with injection of bone)	Gene expression (*Tnfrsf11a*, *Tnfsf11*, *Cys1*, *Cnr2*) Protein levels (CB2, RANKL) OCs number	↑ *Cnr2*, *Tnfrsf11a*, *Tnfsf11*, *Cys1*, CB2, RANKL, OCs number	[58]
50 mg/mL Ti particles (ø < 20 µm)	30 female BALB/c mice (8–10 weeks old). Murine air-pouch model (also with injection of bone)	Gene expression (*Tnfrsf11a*, *Tnfsf11*) Protein levels (RANK, RANKL) Pouch membrane thickness, infiltrated cells Bone erosion OCs number	↑ *Tnfrsf11a*, *Tnfsf11*, RANK, RANKL, bone erosion, pouch membrane thickness, infiltrated cells, OCs number	[59]
50 mg/mL Ti particles (ø < 20 µm)	30 female BALB/c mice (8–10 weeks old). Murine air-pouch model (also with injection of bone)	Pouch membrane thickness, infiltrated cells Bone erosion OCs number Protein levels (MMP9, TNFα, P38 MAPK, p-p38 MAPK) Gene expression (*Mmp9*, *Tnf*)	↑ *Mmp9*, *Tnf*, MMP9, TNFα, p-p38/p-38 MAPK, pouch membrane thickness, bone erosion, infiltrated cells, OCs number	[60]
10 mg/mL Ti particles (ø 4.50 µm)	20 female BALB/c mice (6–8 weeks old). Murine air-pouch model (also with injection of bone)	Pouch membrane thickness Infiltrated cells Protein levels (IL1β, TNFα) Gene expression (*Il1*, *Tnf*)	↑ Edema, pouch membrane thickness, infiltrated cells, IL1β, TNFα, *Il1*, *Tnf*	[33]
10 mg/mL metallic wear particles from the stem of hip prosthesis mainly containing Ti, Co, Cr (ø < 6.67 µm)	18 male BALB/c mice (8 weeks old). Murine air-pouch model	Pouch membrane thickness Infiltrated cells Gene expression (*Tnf*, *Il1*, *Nfatc1*) Protein levels (IL1β, TNFα, NFκB /p65)	↑ Pouch membrane thickness, infiltrated cells, *Tnf*, *Nfatc1*, *Il1*, NFκB /p65, TNFα, IL1β	[56]
30 mg/mL Ti particles (ø < 3.6 µm)	14 C57BL/6J male mice (6–7 weeks old). Murine calvarial model	BV/TV, total porosity OCs number Bone resorption Fibrous tissue Gene expression (*Tnfsf11*) Protein levels (RANKL)	↓ BMD, BV, BV/TV; ↑ OCs number, *Tnfsf11*, RANKL, bone resorption, fibrous tissue	[53]
300 mg/mL Ti particles (ø 0.52 µm)	14 C57BL/6J mice (6–7 weeks old). Murine calvarial model	OCs number BV/TV, Total porosity, bone resorption area Gene expression (*Tnfsf11*)	↑ Bone resorption, OCs number, total porosity, *Tnfsf11*; ↓ BV/TV	[64]
20 mg Ti particles (ø < 20 µm)	54 Sprague Dawley rats (12 weeks old). Fluid flow (20 pressure cycles at 0.17 Hz twice a day) or particles induced models	OCs number Gene expression (*Tnf*, *Il1*, *Cx3cl1*, *Nos_2_*, *Tnfrsf11b*, *Tnfrsf11a*, *Ctsk*, *Mmp9*, *Trap*, *Ptges*, *Ccl2*)	Fluid flow: ↑ OCs number, *Ptges*, *Nos_2_*, *Il6*, *Ctsk*. Particles: ↑ OCs number, *Ptges*, *Nos_2_*, *Tnf*, *Il1*, *Ccl2*, *Il6*, *Ctsk*, *Tnfrsf11a*	[65]
20 mg dried PE particles (ø 7.23 µm)	6 C57BL/6 male mice (10 weeks old). Murine calvarial model (0.5 × 0.5 cm^2^ area of periosteum exposed)	BV/TV OCs number Protein levels (ALP, Osterix) Gene expression (*Tnfrsf11a*, *Ctsk*, *Tnf*, *Il6*, *Il1*, *Cyc1*, *Rpl19*)	↑ Osteonecrosis, osteolytic lesions, empty lacunae, ALP, Osterix production, thickening of inflammatory membrane, infiltrated cells, *Tnfrsf11a*, *Ctsk*, *Tnf*, *Il6*, *Il1*	[61]
20 µg PE particles (ø 5.14 µm)	24 C57BL/6J healthy female mice, 24 C57BL/6J OVX female mice (11 weeks old), 24 C57BL/6J OVX female mice + E. Murine calvarial model (0.5 × 0.5 cm^2^ area of periosteum exposed)	BV Tb.Th Bone erosion SSA OCs number Protein levels (IL1b, IL6, TNFa, RANKL) Gene expression (*Tnfrsf11a*, *Tnfsf11*, *Tnfrsf11b*)	In healthy and OVX mice ↑ bone erosion, fibrous and granulomatous scar tissue, SSA, OCs number, RANKL, *Tnfrsf11a*, *Tnfsf11/Tnfrsf11b*; ↓ BV, Tb.Th.	[18]
10 mg PE particles (ø 7 µm)	38 female Balb/c mice (6–8 weeks old). Murine calvarial model (1 × 1 cm^2^ area of periosteum exposed)	BV, BMD, Tb.Th.3D, BS/BV Protein levels (OPN) Gene expression (*Nell1*, *Spp1*, *Tnfsf11*, *Runx2*) Young’s modulus	↓ BV, BMD, Tb.Th.3D, Young’s modulus ↑ BS/BV, bone resorption, *Spp1*, *Tnfsf11*	[63]
0.5, 2, 5 or 10 mg PE particles (ø 7 µm)	61 NFκB/luc tg mice (7–8 weeks old). Murine calvarial model (1 × 1 cm^2^ area of periosteum exposed)	Total influx Gene expression (*Nfκb* *(p100/p52)*, *Tnf*, *Il1*, *Tnfsf11*, *Cox_2_*) OCs number Bone erosion	↑ Fibrous granulomatous tissues, bone resorption, OCs number, total influx, luciferase activity, *Nfκb* *(p100/p52)*, *Tnf*, *Il1*, *Tnsfs11*, *Cox_2_ (*dose-dependent manner)	[62]

**Table 3:** Clinical studies.

Type of Implant	Patients Groups	Evaluations	Results: Osteolysis vs. Control	Ref.
Not specified	(1) Synovial membrane from 15 pz with THR due to fracture of femoral neck (66.9 ± 6.23 years old) (2) Interfacial membrane from 15 pz with osteolysis of failed THR (66.5 ± 7 years old)	Number of FBs, histiocytes; Protein levels (MIF, CD68); Gene expression (*Mif*)	↑ MIF, *Mif*, CD68+ cells, macrophages and FBs number	[67]
Not specified	Pseudosynovial membranes from: (1) pz with TKR due to OA (52–79 years old) (2) pz with THR due to OA (28–68 years old) (3) pz with osteolysis due to failed TKR (56–88 years old) (4) pz with osteolysis due to failed THR (31–86 years old)	Protein levels (RANKL, OPG, IL8, CCL3, DC-STAMP, SOCS3) Gene expression (*Tnf*, *Il6*, *Chit1*, *Ccl18*, *Il8*, *Ccl3*, *Mmp9*, *Dcst2*, *Tnfrsf11b*, *Tnfsf11*, *Bmp4*, *Socs9*, *Nfκb*, *Hprt1*)	↑ *Chit1*, *Il8*, *Mmp9;* ↓ *Tnf*, *Tnfrsf11b*, *Bmp4* in failed TKR for osteolysis ↓ *Ccl3*, *Dcst2* in failed TKR more than in failed hip	[68]
Not specified	Synovial membrane from: (1) 3 healthy pz (12–41 years old) (2) 3 pz with TKR due to OA (71–82 years old) (3) 3 pz with osteolysis due to failed TKR (60–74 years old)	Protein levels (TWEAK, p-MAPK, MAPK) Gene expression (*Tnfsf12*, *Mapk14*)	↑ inflammatory cells, hyperplasia, TWEAK, *Tnfsf12*, p-MAPK in failed TKR for osteolysis more than healthy pz or OA TKR	[19]
Cemented metal on conventional PE bearing couple	Peripheral whole blood from: (1) 356 pz with THR due to OA (65 ± 8 years old) (2) 275 pz with THR due to OA with osteolysis (59 ± 9 years old)	Genotyping (*Md2*, *Msk1*, *Msk2*, *Myd88*, *Nod1*, *Nod2*, *P2y1*, *P2y6*, *P27*, *Sqstm1*, *Tlr1*, *Tlr2*, *Tlr4*, *Tlr5*, *Tlr6*, *Tlr9*, *Tram*, *Tirap*, *Trif*, *Dkk1*, *Kremen2*, *Lrp5*, *Lrp6*, *Sfrp1*, *Sost*, *Wnt3a*, *Tnfrsf11a*, *Tnfrsf11b*, *Tnfsf11*)	SNPs associated with osteolysis susceptibility: 4 within RANK, 1 within KREMEN2, 1 within OPG, 1 within SFRP1, 1 within TIRAP; SNPs associated with time to implant failure: 2 within LRP6, 1 within LRP5, 1 within NOD2, 1 within SOST, 1 within SQSTH1, 1 within TIRAP, 1 within TRAM	[66]
PE particles	(1) Joint capsule and synovium of 12 pz with THR/TKR due to OA (2 male, 10 female; 60–81 years old) (2) Acetabular membrane, femoral membrane, joint capsule of 12 pz with osteolysis due to failed THR/TKR (4 male, 18 female; 60–81 years old)	% area resorbed Gene expression (*Oscar*, *FcRg*, *Trem2*, *Dap12*)	↑ % resorbed area, OCs number, *Oscar*, *FcRg*, *Trem2*, *Dap12* in THR/TKR due to OA more than failed THR/THR for osteolysis	[70]
Cemented stainless steel and cemented all-PE acetabular component	hPBMCs from: (1) 12 pz with THR/TKR due to OA (75 ± 6 years old) (2) 20 pz with osteolysis due to failed THR/TKR (75 ± 6 years old)	Gene expression (*Il1a*, *Il1*, *Il1ra*, *Il6*, *Il10*, *Il18*, *Tnf*)	↑ *Il1a*, *Il1*, *Il6*, *Il10*, *Il18*, *Tnf* in THR/TKR due to OA more than failed THR/THR for osteolysis	[69]

**Table 4:** Schematic summary of results: major influence/modulation of wear particles on inflammation, osteoclastogenesis and osteoblastogenesis related genes.

Particle Types	Studies	Inflammation	Osteoclastogenesis	Osteoblastogenesis
Ti	In vitro studies	↑ *Tnfα, Il6, Il1, Nos_2_, Cox_2_, Nfκb, Mmp9*	↑ *CatK*, *Rank, Rankl, Trap, Nfatc1*	↓ *Tnfrsf11b*
	In vivo studies	*↑ Mmp9, Tnfα, Il1, Cox_2_, Nos_2_, Il6*	*↑ Nfatc1, Trap, Rank, Rankl*	↓ *Tnfrsf11b*
PE	In vitro studies	*↑ Tnfα, Il6, Mmp9*	*↑ Cathepsin K, Rank, Rankl*	↓ *Tnfrsf11b*
	In vivo studies	*↑ Tnfα, Il1, Il6, Cox_2_*	*↑ Rank, Rankl, Cathepsin K*	↓ *Tnfrsf11b*
	Clinical studies	*↑ Il1α, Il1, Il6, Tnfα, Il18*	*↑ Oscar, Trem2, Dap12, Fcrg*	
PMMA	In vitro studies	*↑ Il1, Nfκb*	*↑ Cathepsin K, Rank, Trap*	↓ *Runx2, Osx, Ocn*
Co-Cr	In vitro studies	*↑ Tnf α, Il6*	*↑ Nfatc1, Rankl*	↓ *Tnfrsf11b, Runx2, Osx*

## Abbreviations

ø	Diameter
AKT	Protein kinase B
ALP	Alkaline phosphatase
BAD	Bcl-2-associated death promoter
TpBCL2	B-cell lymphoma 2
*Bglap*	Osteocalcin gene
BMD	Bone mineral density
BMM	Bone marrow monocytes
BMP	Bone morphogenetic protein
BS/BV	Bone surface/bone volume
BV	Bone volume
BV/TV	Bone volume/tissue volume
*Calcr*	Calcitonin receptor gene
CAT	Chloramphenicol acetyltransferase
*Catk*	Cathepsin k gene
CB	Cannabinoid receptor
CCL	C–C motif chemokine ligand
CCR	C–C motif chemokine receptor
CD	Cluster of differentiation
CE	Aluminia ceramic
CGRPR1	Calcitonin gene-related peptide type 1 receptor
CHIT1	Chitinase 1
CHOP	C/EBP homologous protein
CM	Conditioned medium
*Cnr2*	Cannabinoid receptor type 2 gene
COLL I	Collagen type I
COMP	Cartilage oligomeric matrix protein
COX	Cyclooxygenase
CREB	cAMP response element-binding protein
CSF1	Colony stimulating factor 1
CXCL	C-X-C motif chemokine ligand
CXCR	C-X-C motif chemokine receptor
CYS1	Cystin 1
DAP12	TYRO protein tyrosine kinase binding protein
DC-STAMP	Dendritic cells (DC)-specific transmembrane protein
DKK	Dickkopf-related protein
E	Estrogen
ERK	Extracellular signal–regulated kinases
FAM213	Family with sequence similarity 213 Member A
FB	Fibroblasts
FGFR	Receptor of fibroblast growth factor
Gpx	Glutathione peroxidase
GR	Glutathione reductase
HA	Hydroxyapatite
HDP	High density polyethylene
HPRT1	hypoxanthine phosphoribosyltransferase 1
IGF	Insulin-like growth factor
IL	Interleukin
*Il*	Interleukin gene
*Il1ra*	Interleukin 1 receptor antagonist gene
iNOS	Inducible nitric oxide synthase
IRE-1	Inositol-requiring kinase 1
KREMEN1	Kringle containing transmembrane
LDH	Lactate dehydrogenase
*Lrp5*	Low-density lipoprotein receptor-related protein 5 gene
MAPK	Mitogen-activated protein kinase
MCP-1	Monocyte chemoattractant protein-1
MD2	Myeloid differentiation protein-2
MhcII	Major histocompatibility complex class II
MIP-1α	Macrophage inflammatory protein-1 alpha
MMP	Matrix metalloprotease
MRC	Mannose receptor C
MSC	Mesenchymal stem cells
MSK	Mitogen- and stress-activated protein kinase
*Myd*	Myeloid differentiation primary response gene
Nell1	Neural EGFL like 1
*Nfatc1*	Nuclear factor of activated T-cells, cytoplasmic 1 gene
*Nfκb*	Nuclear factor κ-B
NO	Nitric oxide
NOD	Nodulation factor
NP	Nanoparticles
OA	Osteoarthritis
OB	Osteoblasts
OC	Osteoclasts
OCN	Osteonectin
OPG	Osteoprotegerin
OPN	Osteopontin
OSCAR	Osteoclast-associated immunoglobulin-like receptor
*Vegf*	Vascular endothelial growth factor gene
P2RY1	Purinergic receptor P2Y1
P2RY6	Pyrimidinergic receptor P2Y6
*Pdgf*	Platelet derived growth factor gene
PGE_2_	Prostaglandin E2
PIK3CB	Phosphatidylinositol-4,5-bisphosphate 3-kinase catalytic subunit beta
PIK3R2	Phosphoinositide-3-kinase regulatory subunit
PMMA	Poly methyl methacrilate
*Ptges*	Prostaglandin E synthase gene
pz	Patients
RANK	Receptor activator of nuclear factor κ-B
RANKL	Receptor activator of nuclear factor κ-B ligand
ROS	Reactive oxygen species
*Rpl19*	Ribosomal protein L19 gene
*Runx2*	Runt-related transcription factor 2 gene
SF	Synovial fibroblasts
SFRP1	Secreted frizzled related protein
SOCS3	Suppressor of cytokine signaling 3
SOD	Superoxide dismutase
SOST	Sclerostin
SOX9	Sry-related HMG box
*Sp7*	Osterix gene
SPP1	Secreted phosphoprotein 1
SQSTM1	Sequestosome 1
SSA	Sagittal suture area
TAK	Transforming growth factor beta-activated kinase
Tb.Th	Trabecular thickness
THA	Total hip arthroplasty
THR	Total hip replacement
Ti	Titanium
*Ticam*	Toll-like receptor adaptor molecule gene
TiO_2_-M	Micro-sized titanium dioxide particles
TiO_2_-N	Nano-sized titanium dioxide particles
TIRAP	TIR domain containing adaptor protein
*Tlr*	toll-like receptor gene
*Tnfrsf*	TNF receptor superfamily gene
*Tnfsf11*	*RANKL* gene
*Tnfrsf11b*	*Osteoprotegerin* gene
*Tnfrsf11a*	*RANK* gene
TNFα	Tumor necrosis factor α
*Tnf*	Tumor necrosis factor α gene
TKR	Total knee replacement
TRAF	TNF receptor associated factor
TRAM	Translocation associated membrane protein
*Trap*	Tartrate-resistant acid phosphatase gene
*Trem*	Triggering receptor expressed on myeloid cells gene
*Trif*	TIR-domain-containing adapter-inducing interferon-β gene
UHMWPE	Ultra high molecular weight polyethylene
